# Passive hyperthermia increases blood circulation in specific regions, largely independent of conduit artery mechanics and cardiac performance

**DOI:** 10.1113/EP093331

**Published:** 2026-02-15

**Authors:** Nuno Koch Esteves, Kazuhito Watanabe, Francesca R. Cavallo, Ashraf W. Khir, José González‐Alonso

**Affiliations:** ^1^ Department of Sport, Health and Exercise Sciences, College of Health, Medicine and Life Sciences Brunel University of London Uxbridge UK; ^2^ ThermosenseLab, Skin Sensing Research Group, School of Health Sciences University of Southampton Southampton UK; ^3^ Faculty of Education and Human Studies Akita University Akita Japan; ^4^ Department of Epidemiology and Biostatistics, School of Public Health Imperial College London London UK; ^5^ Department of Engineering Durham University Durham UK

**Keywords:** arterial compliance, circulation, hyperthermia, wave intensity analysis

## Abstract

Passive hyperthermia increases net peripheral and systemic blood flow in humans and other animals, yet the underlying haemodynamic forces that selectively accelerate blood movement remain incompletely characterized. Wave intensity analysis offers insight into the respective contributions of the heart and the vascular system to changes in blood circulation during physiological stress; however, the specific impact of hyperthermia on wave intensity metrics has not been elucidated comprehensively. To address this, we investigated wave speed and wave intensity parameters in the common carotid artery, along with local arterial distensibility in the internal carotid, brachial and common femoral arteries, in addition to total arterial compliance, in eight healthy males across four protocols: (1) 3 h of control measurements in normothermic conditions; (2) 3 h of one‐leg heating; (3) 3 h of two‐leg heating; and (4) 2.5 h of whole‐body heating. Forward compression (1.5‐fold; *P *= 0.041) and forward expansion (5.2‐fold; *P *< 0.0001) waves in the common carotid artery (indices of ventricular contractility and late‐systolic blood flow deceleration, respectively) increased exclusively during whole‐body heating. In contrast, backward compression waves, wave speed, distensibility and reflection index remained unaltered across all conditions. Notably, distensibility in the major conduit arteries perfusing the brain (internal carotid artery), forearm (brachial artery) and leg (common femoral artery), in addition to total arterial compliance, remained unchanged across all conditions. Collectively, these findings suggest that increases in blood circulation within specific regions of the human body during passive hyperthermia are largely independent of conduit artery mechanics and cardiac performance.

## Abstract (highlights)

- **What is the central question of the study?**
  What are the contributions of peripheral and cardiac haemodynamic forces to changes in blood circulation induced by lower‐limb and whole‐body hyperthermia?
- **What is the main finding and its importance?**
  In the common carotid artery, forward compression and expansion waves increased only during whole‐body heating, while other wave properties and distensibility were unchanged. Distensibility in the main brain, forearm and leg conduit arteries, in addition to total arterial compliance, remained unaffected across conditions. Thus, the increase in total blood flow during passive hyperthermia is achieved without significant elastic adjustments in the conduit arteries.

## 1 INTRODUCTION

Passive whole‐body hyperthermia significantly impacts human haemodynamics by increasing limb tissue, myocardial and systemic blood flow (${\dot{Q}}_{\mathrm{sys}}$), while reducing perfusion in the renal and splanchnic circulations. Concurrently, arterial pressure and cerebral blood flow decline slightly or remain unchanged, and central venous pressure decreases (Barry et al., 2024; Chiesa et al., 2016; Gibbons et al., 2021, 2020; Leaney et al., 2025; Meade et al., 2025; Ogoh et al., 2013; Pearson et al., 2011; Rowell et al., 1970, 1969; Stöhr et al., 2011; Watanabe et al., 2024). Traditionally, central haemodynamic forces evoked by the pressure‐generating function of the heart are seen as the primary drivers for changes in blood circulation (Rowell, 1993; Secomb, 2016). Specifically, Rowell proposed that during direct heating, heat‐induced cutaneous vasodilatation substantially elevates total vascular conductance, thereby necessitating a marked increase in ${\dot{Q}}_{\mathrm{sys}}$, achieved predominantly through tachycardia and modest increases in left ventricular (LV) contractility, together with a redistribution of blood flow from central and visceral circulations (e.g., splanchnic and renal) to the cutaneous vasculature to preserve arterial pressure and enable heat loss (Rowell, 1974, 1986). The regulatory prioritization of arterial blood pressure and cardiac performance underpins the view that peripheral tissue perfusion is primarily driven by the heart. Recent findings call into question this prevailing paradigm, revealing that selective increases in blood circulation during one‐leg, two‐leg and whole‐body passive hyperthermia are largely unrelated to alterations in central haemodynamic forces, LV function or respiratory and metabolic activity (Watanabe et al., 2024). This indicates that such factors are not essential to elevate ${\dot{Q}}_{\mathrm{sys}}$ (Watanabe et al., 2024). Rather, Watanabe et al. (2024) postulated that the peripheral heat‐related changes in the velocity and kinetic energy of flowing blood (and, consequently, venous flow to the heart) play a significant role in aligning peripheral and central circulations (Furst & González‐Alonso, 2025; Koch Esteves et al., 2021, 2024). However, it remains unknown how hyperthermia affects the haemodynamic forces underlying the changes in blood velocity and kinetic energy of blood returning to the heart.

Wave intensity analysis (WIA) provides insight into the interplay between the heart and the vascular system, particularly the elasticity of vessels. WIA decomposes the dynamic forces driving blood flow within arteries by separating the forward‐ and backward‐travelling waves. The typical output includes three wave peaks: the forward compression wave (FCW); the forward expansion wave (FEW); and the backward compression wave (BCW) (Pomella et al., 2018). These waves are indicative of LV contractility during early systole, late‐systolic flow deceleration in late systole, and reflected waves returning to the left ventricle in mid‐systole, respectively (Pomella et al., 2018). Consequently, WIA might further elucidate the relative contributions of peripheral and central mechanisms to hyperthermia‐induced limb and systemic hyperaemia. Current evidence exploring changes in WIA metrics during passive hyperthermia suggests no differences following 10 min of hot‐water immersion (Hatano et al., 2002), 30 min of upper‐ or lower‐limb heating (Athaide et al., 2023) or 3 h of one‐leg heating (Koch Esteves et al., 2021). This might be attributed to the thermal stimuli not being sufficient to induce central changes in cardiac dynamics, because increases in LV contractility have been observed during whole‐body heating (Brothers et al., 2009; Crandall et al., 2008; Foster et al., 2025; Nelson et al., 2010; Watanabe et al., 2024), but not during one‐ or two‐leg heating, when internal hyperthermia levels were relatively low (Watanabe et al., 2024). However, it remains to be investigated whether high levels of hyperthermia alter WIA metrics.

Wave speed serves as an indirect indicator of local arterial distensibility, which is colloquially synonymous with arterial compliance (the reciprocal of stiffness) (Feng & Khir, 2010; Hughes et al., 2013; Mynard et al., 2018; Parker & Jones, 1990). The distinction lies in that distensibility is a normalized, relative measure that enables comparison between vessels of different sizes, whereas compliance represents an absolute, size‐dependent measure. Arterial distensibility reflects the ability of arteries to expand and contract with cardiac pulsation and relaxation (Godia et al., 2007; Kawasaki et al., 1987) and offers insight into the haemodynamic forces that modulate blood circulation. Fluctuations in local arterial blood volume reflect changes in blood velocity and distensibility/compliance, both influenced by arterial diameter and pressure variations throughout the cardiac cycle (Nichols et al., 2022). Alterations in pulse pressure, in turn, reflect changes in stroke volume ejected during systole, in addition to the compliance and resistance of the arterial system, which together influence diastolic pressure (Nichols et al., 2022). Evidence indicates that heat can influence these vascular properties in a region‐specific manner. Regional leg heating selectively elevates blood velocity and flow in heated tissues without altering perfusion pressure (Koch Esteves et al., 2024). Whole‐body heating increases blood flow in the femoral arterial and venous systems, including the saphenous vein, in humans (Chiesa et al., 2016). Likewise, local infrared radiation can increase blood velocity in the vitelline vessel of the 3‐day‐old chick embryo by 3.7‐fold, even in the absence of cardiac activity (Li & Pollack, 2023). Heating also modulates vascular tone in skeletal muscle, gut, brain and skin arteries, markedly increasing arterial and venous blood flow in the radial muscle branch artery and a neighbouring vein in the mouse forelimb (Phan et al., 2025). Collectively, these findings suggest that heat‐mediated elevations in the kinetic energy of blood returning to the heart might involve region‐specific changes in conduit artery mechanics, including passive properties, such as arterial distensibility/compliance, in addition to active modulation through vascular smooth muscle tone.

The current literature, however, indicates that central and peripheral arterial stiffness or distensibility is generally unchanged during acute hyperthermia (Ganio et al., 2011; Moyen et al., 2016; Schlader et al., 2019), although stiffness may decline following cessation of heating (Lee et al., 2018; Sugawara & Tomoto, 2021; Thomas et al., 2017). In mice, temperature modulates myogenic tone in peripheral arteries via the thermosensitive channels TRPV1 and TRPM4, paradoxically increasing limb tissue perfusion during heating despite reduced vessel diameter (Phan et al., 2025). To date, no study has investigated comprehensively the effects of lower‐limb or whole‐body hyperthermia on arterial distensibility/compliance; whether it be employing the velocity–area ln(*D*)*U*‐loop technique (Borlotti et al., 2012; Feng & Khir, 2010) or pulse pressure–diameter‐based methods (Nichols et al., 2022; Parker & Jones, 1990). Understanding how passive hyperthermia affects local arterial distensibility and total arterial compliance in humans might provide insight into a heat‐activated flow‐driving mechanism that accelerates venous return and ultimately enhances ${\dot{Q}}_{\mathrm{sys}}$.

This study aimed to explore the haemodynamic forces governing blood circulation during control normothermia and three levels of passive hyperthermia (one‐leg, two‐leg and whole‐body heating) using WIA and estimates of regional (head, brain, forearm and leg) distensibility and total arterial compliance. We sought to determine whether peripheral and/or heart‐derived haemodynamic forces, as reflected by wave intensity (WI)‐derived parameters, local arterial distensibility and total arterial compliance responses, contribute to the increase in ${\dot{Q}}_{\mathrm{sys}}$ during hyperthermia. We hypothesize that passive thermal interventions enhance ${\dot{Q}}_{\mathrm{sys}}$ in part via a vessel‐based, non‐cardiogenic mechanism. Specifically, thermally induced alterations in vascular tone and/or vasodilatation can enhance perfusion and accelerate venous return to the heart, independently of conduit artery mechanics and cardiac performance.

## 2 MATERIALS AND METHODS

### 2.1 Ethical approval

The study was approved by the Brunel University of London Research Ethics Committee (6237‐A‐Jun/2017‐7569‐2) and was carried out in accordance with the *Declaration of Helsinki*. Written informed consent was obtained from all participants prior to commencement of the study.

### 2.2 Participants

A convenience sample of eight healthy males, with a mean (±SD) age of 29 ± 11 years, height of 179 ± 7 cm and body mass of 73 ± 10 kg, participated in the study. The participants were non‐smokers and were not taking prescription medications. Participants self‐reported themselves as physically active, regularly participating in physical activity three to six times per week.

### 2.3 Experimental design

The present study was part of a larger investigation evaluating human circulatory control during hyperthermia, the procedures of which are described in detail elsewhere (Watanabe et al., 2024). The present study reports new WIA and regional and total arterial compliance data to provide further insights into the control of circulation during hyperthermia. Participants visited the laboratory on four occasions separated by >3 days, undergoing four randomly assigned and counterbalanced protocols: (1) 3 h of no heating (control); (2) 3 h of one‐leg heating; (3) 3 h of two‐leg heating; and (4) 2.5 h of whole‐body heating. In comparison to the first three protocols, the whole‐body heating trial was terminated earlier because all participants reached their limit of heat tolerance after 2.5 h (apart from one participant who terminated at 2 h). For all four protocols, participants were required to abstain from strenuous exercise and alcohol intake for 24 h and caffeine consumption for 12 h before the commencement of the protocol.

On the experimental day, participants arrived at the laboratory postprandial at 08.00 h, and body mass was measured after the voiding of urine. Breakfast prior to the trials was not standardized; however, participants were instructed to consume their typical breakfast at their usual preferred time. The participants then entered an environmental chamber set at 23°C and rested in a supine position on a bed. Following instrumentation and baseline measurements prior to heating, participants were fitted with a water‐perfused garment on their right leg for protocol 2 (one‐leg heating), both legs for protocol 3 (two‐leg heating) and both legs and upper body except the head for protocol 4 (whole‐body heating). The garment was connected to a thermostatically controlled water circulator (F‐34, Julabo, Germany), which continuously circulated hot water (50°C outlet temperature exiting the water circulator system), and was wrapped in a survival blanket to limit heat loss.

### 2.4 Measurements

During the experimental protocols, LV volumes and functions and common carotid artery (CCA), internal carotid artery (ICA), brachial artery (BA) and common femoral artery (CFA) haemodynamics were assessed every 30 min using an ultrasound system (see below for further details). Heart rate, blood pressure and body temperatures were recorded continuously. Participants ingested fluids during the heating protocols to maintain a normal hydration status (i.e., 0.1 ± 0.2, 0.3 ± 0.1 and 0.9 ± 0.1 L h^−1^ of water during one‐leg, two‐leg and whole‐body heating, respectively).

### 2.5 Limbs and head haemodynamics

#### 2.5.1 Arterial diameter, blood velocity and blood flow

Vascular ultrasound assessments were collected every 30 min during the protocols in the CCA, ICA, BA and CFA, as previously described (Watanabe et al., 2024), using an ultrasound system (VIVID 7 Dimension; GE Medical, Horton, Norway) equipped with a 10 MHz linear probe (10L, GE Healthcare). Right CCA images were recorded ∼1.5 cm proximal to the carotid bifurcation. Longitudinal images of the arteries were recorded when the intima–media boundary was clearly visible. The CCA diameter was determined using CAROLAB (Zahnd et al., 2013). CAROLAB uses block matching to provide an accurate measurement of the vessel diameter at each frame. Alongside the diameter waveforms, this technique outputs time‐averaged mean diameter (*D*_mean_), minimum diameter during diastole (*D*_min_) and maximum diameter during systole (*D*_max_). The CCA blood velocity was measured simultaneously with artery images using continuous pulsed‐wave Doppler at a frequency of 4.4 MHz, with an insonation angle consistently <60° and the sample volume extended to cover the entire vessel lumen. Continuous 12 s blood velocity profiles were recorded and analysed offline in MATLAB (version R2023b, The MathWorks, Inc., Natick, MA, USA), which extracted the flow velocity waveforms, using custom‐designed algorithms, as previously reported (Koch Esteves et al., 2021; Negoita et al., 2018). Accompanying the velocity waveforms, this code provided *D*_mean_, *D*_min_ and *D*_max_. The ICA, BA and CFA diameter and velocity were calculated using the manufacturer's software (EchoPAC PC v.112; GE Healthcare, Chalfont Saint Giles, UK), as previously reported (Watanabe et al., 2024).

The CCA, ICA, BA and CFA blood flow (BF) were calculated using the following equation: $\mathrm{BF}=U_{\mathrm{mean}}\times \pi \times {\left(\frac{D_{\mathrm{mean}}}{2}\right)}^{2}\times 60$, where BF is represented as litres per minute, *U*_mean_ is the average blood velocity (in centimetres per second) and *D*_mean_ is the average diameter (in centimetres). Additionally, vascular conductance (VC) was calculated using the following formula: VC=BF/MAP, where it is represented as millilitres per minute per millimetre of mercury, BF is blood flow (in millilitres per minute), and MAP is the mean arterial pressure (in millimetres of mercury). Measurement of arterial pressure is discussed in full below.

#### 2.5.2 Wave intensity, local arterial distensibility and total arterial compliance

After obtaining the ultrasound B‐mode scans, as described above, images were exported as DICOM files for offline analysis. Wave speed determination and WIA were performed on the CCA. WIA was conducted at all 30 min time points for the CCA. Diameter waveform extraction was performed using CAROLAB (Zahnd et al., 2013), as previously described. Extracted diameter waveforms were saved as Excel files (Microsoft Corporation, Redmond, WA, USA) for later analysis. Doppler ultrasound DICOM files were analysed in MATLAB (v.R2023b, The MathWorks, Inc., Natick, MA, USA) to extract the flow velocity waveforms, as previously described.

This diameter and flow velocity waveforms were then used to calculate wave speed (c) using the ln(*D*)*U*‐loop method (Feng & Khir, 2010); the following equation was used: $c=\pm \frac{1}{2}\frac{dU_{\pm }}{d{\left(\ln D\right)}_{\pm }}$ where dU and d(lnD) are the incremental differences between adjacent data of velocity (U) and diameter (D). Moreover, peak FCW, peak FEW and peak BCW, which reflect LV contractility, late‐systolic flow deceleration and downstream reflections, respectively, were calculated using previously documented techniques (Pomella et al., 2018). Data outputs were averaged over two scans for the same time point, with three waveforms analysed per scan. Subsequently, with the determination of c, distensibility $\left(D_{s}\right)$ was calculated using the following Bramwell & Hill (1922) equation: $D_{s}=p^{-1}\times c^{-2}$, where p represents blood density, which was assumed equal to 1050 kg m^−3^ (Pomella et al., 2018). Moreover, the reflection index (RI) was calculated as the modulus of the ratio of peak BCW to that of FCW (Borlotti et al., 2012).

Furthermore, for the ICA, BA and CFA, where WIA did not occur, local arterial distensibility (10^−3^ mmHg^−1^) was calculated as $D_{s}=\frac{1}{D_{\min}}\times \frac{2\Delta D}{\Delta P}$, where *D*_min_ is diastolic diameter (in centimetres), Δ*D* is the difference between systolic and diastolic diameter (in centimetres), and Δ*P* is pulse pressure (in millimetres of mercury; calculated as the difference between systolic and diastolic blood pressure, respectively). Direct pulse pressure measurements were obtained; thus, upper‐arm blood pressure was used as a surrogate. Total arterial compliance (in millilitres per millimetre of mercury) was calculated as follows: totalarterialcompliance=SV/ΔP, where SV is stroke volume (in millilitres) and Δ*P* is pulse pressure (in millimetres of mercury).

### 2.6 Arterial pressure, cardiac output, vascular conductance and body temperatures

Arterial blood pressure was measured non‐invasively using finger photoplethysmography (Finometer, Finapres Medical Systems, The Netherlands). The monitoring cuff was placed around the middle finger of the right hand, with the forearm and hand supported such that the cuff was positioned at the vertical level of the heart. The MAP was measured by integrating the continuous blood pressure waveform over a 5 min period using data analysis software (LabChart 8, ADInstruments, Australia). The same software was used to determine systolic (SBP) and diastolic (DBP) blood pressures, defined as the highest and lowest values of the pressure waveform, respectively. Heart rate (HR) was monitored via a three‐lead ECG. Systemic BF (${\dot{Q}}_{\mathrm{sys}}$) was calculated as follows: ${\dot{Q}}_{\mathrm{sys}}$ = HR × SV, where HR is heart rate (in beats per minute) and SV is stroke volume, which was measured directly as the difference in LV end‐diastolic and end‐systolic volumes through echocardiographic measurements, as previously reported by Watanabe et al. (2024). Systemic vascular conductance (SVC; in millilitres per minute per millimetre of mercury) was calculated using the following formula: SVC = ${\dot{Q}}_{\mathrm{sys}}$ /MAP, where ${\dot{Q}}_{\mathrm{sys}}$ is cardiac output (in millilitres per minute), and MAP is the mean arterial pressure (in millimetres of mercury). Core temperature was assessed using a commercially available rectal probe (RET‐1, Physitemp Instruments, USA) inserted 15 cm past the sphincter muscle and connected to a thermocouple meter (TC‐2000, Sable Systems, USA). Skin temperature from six sites (forehead, chest, arm, thigh, calf and foot) was obtained using commercially available thermistors (IT‐18, Physitemp Instruments, USA). Mean skin temperature from four sites (chest, arm, thigh and calf) were calculated using a standard weighted formula (Ramanathan, 1964), then mean body temperature was obtained from core and mean skin temperatures, as previously reported (Hardy et al., 1938). The temperature probe and thermistors were connected to a thermocouple meter (TC‐2000, Sable Systems, USA), with the data being sampled at 1000 Hz together with analog signals of the ECG and blood pressure waveform using a data acquisition unit (Powerlab 16/30, ADInstruments, Australia) and analysed offline using a data analysis software (LabChart v.8, ADInstruments, Australia).

### 2.7 Statistical analysis

Data are reported as the mean ± SD. Differences in measured variables were assessed using ANOVA with linear mixed‐effects models. Linear mixed models are more appropriate for the analysis of nested and crossed structures of the data, where there are multiple observations within a single subject in each condition, in addition to multiple observed conditions for each subject (Boisgontier & Cheval, 2016). The random‐effects structure of the mixed‐effect models was determined using theoretical considerations. The model used includes time, protocol and their interaction as fixed effects, and a random intercept accounts for the variation between the baselines of participants. After conducting the mixed‐effects ANOVAs, *post hoc* tests were conducted for significant time × protocol interactions only, using a Bonferroni correction. Values of *P* < 0.05 were considered significant. The strength of the relationships between CCA FCW and indexes of myocardial systolic function were assessed with mixed‐effects models applied to the pooled data from all four trials. Values of *P* < 0.05 were considered significant. Statistical analyses were performed using R v.4.0.5 in RStudio v.1.2.5033 and Python v.3.9.7.

## 3 RESULTS

### 3.1 Body temperatures

All temperatures remained unchanged during the control trial. According to the design, leg skin temperatures increased rapidly by 8°C–10°C in all heating trials, whereas core temperature increased gradually by 0.4 (0.2)°C, 0.7 (0.2)°C and 2.3 (0.4)°C during one‐leg, two‐leg and whole‐body heating, respectively (all *P* < 0.001 for the end of heating vs. respective baseline). Forehead skin temperature remained stable in the control, one‐leg and two‐leg heating trials (*P* = 0.449, *P* = 0.057 and *P* = 1.000 for baseline vs. end of heating, respectively) but increased gradually by 3.2 (1.0)°C with whole‐body heating (*P* < 0.001 at the end of heating).

### 3.2 Peripheral and systemic haemodynamics

**FIGURE 1:**
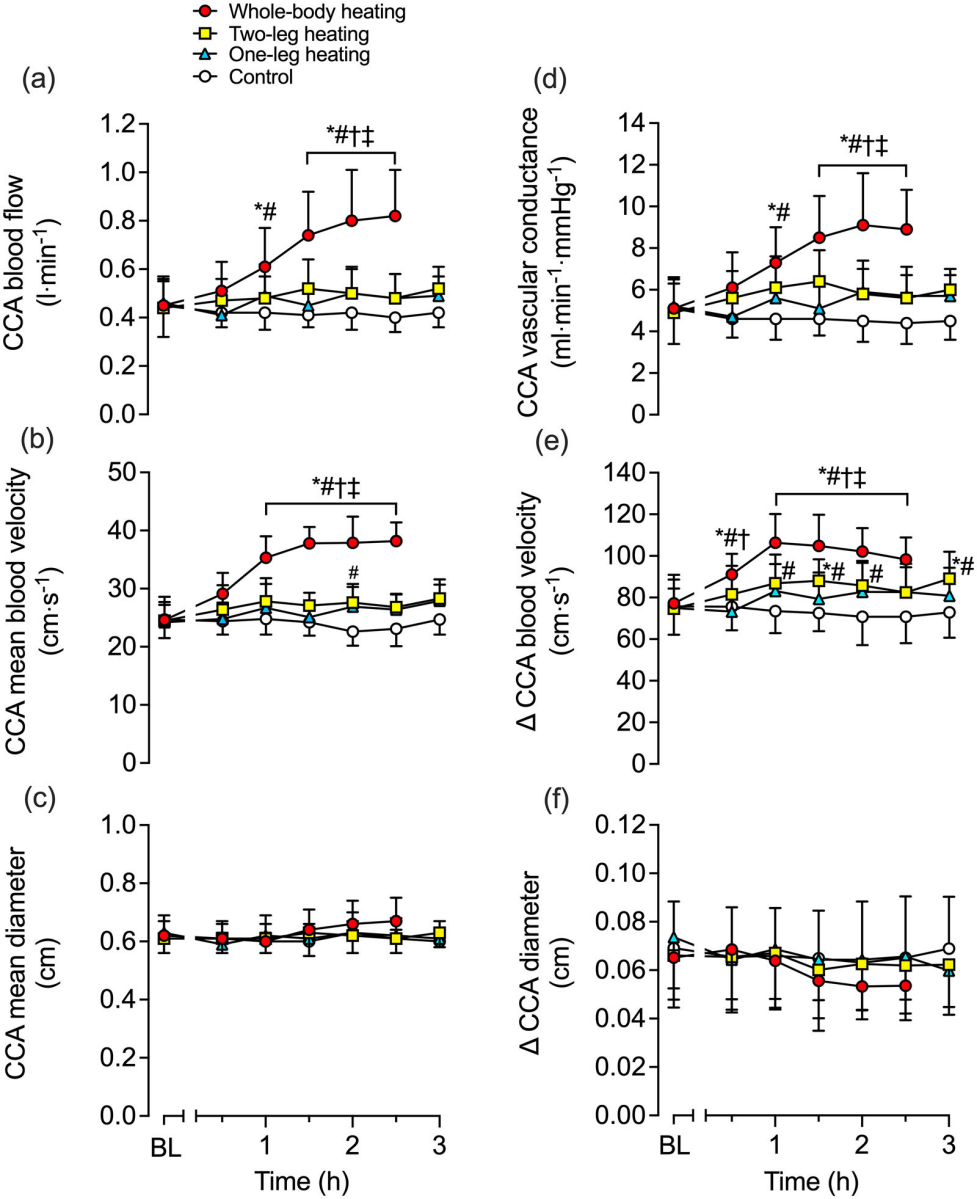
Common carotid artery (CCA) haemodynamics during whole‐body, two‐leg and one‐leg heating and control trials. Data are represented as the mean (SD) for eight participants. Abbreviations: BL, baseline; ∆ CCA blood velocity, difference between the maximal and minimal blood velocities; ∆ CCA diameter, difference between the maximal and minimal diameters. ^*^ *P* < 0.05 versus baseline; ^#^ *P* < 0.05 versus control; ^†^ *P* < 0.05 versus one‐leg heating; ^‡^ *P* < 0.05 versus two‐leg heating.

The CCA BF, ${\dot{Q}}_{\mathrm{sys}}$ and MAP remained stable in control conditions (Figure 1; Table 1). As previously reported, leg (CFA) BF increased progressively in all heating trials up to 0.5–0.9 L min^−1^ above baseline (Watanabe et al., 2024). In contrast, CCA BF increased only during whole‐body heating [+0.382 (0.149) L min^−1^; *P* < 0.0001 vs. baseline at 2.5 h] and was higher compared with control [+0.427 (0.137) L min^−1^], one‐leg heating [+0.347 (0.113) L min^−1^) and two‐leg heating [+0.365 (0.124) L min^−1^] (all *P* < 0.0001; Figure 1). This increase in CCA BF during whole‐body heating was associated with parallel elevations in CCA blood velocity [+13.5 (3.3) cm s^−1^; *P *< 0.0001 vs. baseline], with CCA diameter remaining unchanged (*P* = 0.6626; Figure 1). CCA ∆*U* increased following 3 h of two‐leg heating [+14.4 (10.0) cm s^−1^; *P* = 0.0152 vs. baseline] and 2.5 h of whole‐body heating [+22.6 (14.8) cm s^−1^; *P* < 0.0001 vs. baseline; Figure 1], associated with proportional increases in CCA maximal blood velocity (*U*_max_; *P* < 0.0001; Table 2), while CCA ∆*D* remained unchanged in all protocols (Figure 1). ${\dot{Q}}_{\mathrm{sys}}$ increased gradually with one‐leg, two‐leg and whole‐body heating [+2.0 (0.9), +2.9 (0.9) and +6.7 (1.1) L min^−1^ vs. baseline, respectively; *P* < 0.001; Table 1]. MAP remained stable throughout all protocols (all *P* = 1.000 vs. baseline), but arterial pulse pressure was reduced after 2 h of whole‐body heating compared with baseline and corresponding values during one‐leg heating (Table 1).

**TABLE 1:** Cardiac output, arterial blood pressure, pulse pressure and systemic vascular conductance responses during whole‐body, two‐leg and one‐leg heating and control trials.

	Time (h)									
Parameter		Baseline	0.5	1	1.5	2	2.5	3	Factor	*P*‐value
Cardiac output, L min^−1^										
	Whole‐body heating	5.8 (0.6)	8.9 (1.8)^*^^#†‡^	10.7 (2)^*^ *^#^* ^†‡^	11.7 (2)^*^ *^#^* ^†‡^	12.2 (2.2)^*^ *^#^* ^†‡^	12.4 (1.5)^*^ *^#^* ^†‡^	–	Time	<0.0001
	Two‐leg heating	5.7 (0.9)	6.8 (1)^#^	7.6 (1.3)^*^ *^#^* ^†^	8.1 (1.2)^*^ *^#^* ^†^	8.1 (1.1)^*^ *^#^*	8.3 (1.4)^*^ *^#^*	8.6 (1.3)^*^ *^#^* ^†^	Condition	<0.0001
	One‐leg heating	5.3 (0.6)	5.9 (0.9)	6.3 (0.8)	6.4 (0.9)^#^	6.9 (0.8)^*^ *^#^*	7.0 (0.8)^*^ *^#^*	7.3 (0.8)^*^ *^#^*	Interaction	<0.0001
	Control	5.4 (0.8)	5.3 (0.6)	5.3 (0.7)	5.1 (0.5)	5.2 (0.5)	5.1 (0.7)	5.2 (0.7)		
Mean arterial pressure, mmHg										
	Whole‐body heating	90 (7)	83 (8)^#^	83 (2)	87 (2)	88 (3)	91 (5)	–	Time	0.1046
	Two‐leg heating	90 (7)	84 (6)	81 (6)^#^	81 (5)^#^	86 (4)	86 (4)	88 (5)	Condition	<0.0001
	One‐leg heating	89 (7)	88 (6)	90 (8)	89 (10)	85 (4)	85 (4)	87 (5)	Interaction	0.0018
	Control	90 (6)	93 (7)	92 (6)	91 (6)	95 (8)	91 (6)	94 (7)		
Pulse pressure, mmHg										
	Whole‐body heating	57 (11)	52 (9)	47 (8)	45 (9)	44 (8)^*^^†^	44 (9)^*^^†^	–	Time	0.3212
	Two‐leg heating	55 (9)	55 (10)	54 (7)	54 (8)	54 (8)	53 (8)	54 (8)	Condition	<0.0001
	One‐leg heating	53 (10)	51 (11)	56 (10)	57 (8)	58 (6)	58 (6)	59 (7)	Interaction	0.0026
	Control	56 (7)	55 (9)	54 (10)	53 (11)	52 (11)	50 (11)	50 (12)		
Systemic vascular conductance, mL min mmHg^−1^										
	Whole‐body heating	64 (9)	109 (25)^*^ *^#^* ^†‡^	127 (22)^*^ *^#^* ^†‡^	134 (23)^*^ *^#^* ^†‡^	138 (26)^*^ *^#^* ^†‡^	137 (19)^*^ *^#^* ^†‡^	–	Time	<0.0001
	Two‐leg heating	65 (13)	81 (15)*^#^*	96 (23)^*^ *^#^* ^†^	100 (20)^*^ *^#^* ^†^	94 (16)^*^ *^#^*	96 (16)^*^	99 (17)^*^	Condition	<0.0001
	One‐leg heating	60 (8)	68 (13)	70 (11)	73 (14)	81 (12)^*^ *^#^*	82 (10)^*^ *^#^*	84 (11)^*^ *^#^*	Interaction	<0.0001
	Control	62 (12)	57 (10)	58 (10)	56 (9)	55 (9)	57 (10)	56 (9)		
Total arterial compliance, mL mmHg^−1^										
	Whole‐body heating	2.1 (0.5)	2.5 (0.7)	2.8 (0.6)	2.7 (0.6)	2.8 (0.8)	2.7 (0.8)	–	Time	0.0047
	Two‐leg heating	2.1 (0.5)	2.3 (0.6)	2.3 (0.5)	2.4 (0.6)	2.4 (0.6)	2.5 (0.7)	2.5 (0.7)	Condition	<0.0001
	One‐leg heating	2.1 (0.6)	2.4 (0.9)	2.2 (0.6)	2.1 (0.6)	2.1 (0.5)	2.1 (0.5)	2.1 (0.5)	Interaction	0.0866
	Control	2.0 (0.5)	2.0 (0.6)	2.1 (0.6)	2.1 (0.6)	2.2 (0.7)	2.3 (0.8)	2.3 (0.9)		

*Note*: Data are represented as the mean (SD) for eight participants. The *P*‐values were determined using two‐way ANOVA with repeated measures and the Bonferroni *post hoc* procedure.

* *P* < 0.05 vs. baseline.

^#^ *P* < 0.05 vs. control.

^†^ *P* < 0.05 vs. one‐leg heating.

^‡^ *P* < 0.05 vs. two‐leg heating.

**TABLE 2:** Common carotid artery velocity and diameter responses during whole‐body, two‐leg and one‐leg heating, and control trials.

Parameter		Time (h)								
		Baseline	0.5	1	1.5	2	2.5	3	Factor	*P*‐value
*U*_max_, m s^−1^										
	Whole‐body heating	0.77 (0.14)	0.91 (0.10)^*^, ^#^, ^†^, ^‡^	1.07 (0.14)^*^, ^#^, ^†^, ^‡^	1.05 (0.15)^*^, ^#^, ^†^, ^‡^	1.02(0.11)^*^, ^#^, ^†^, ^‡^	0.98 (0.11)^*^, ^#^, ^†^, ^‡^	–	Time	<0.0001
	Two‐leg heating	0.75 (0.10)	0.82 (0.14)	0.87 (0.14)^#^	0.88 (0.10)^#^	0.86 (0.12)^#^	0.83 (0.12)	0.89 (0.13)^*^, ^†^	Condition	<0.0001
	One‐leg heating	0.75 (0.14)	0.73 (0.09)	0.83 (0.13)	0.79 (0.13)	0.83 (0.14)	0.83 (0.14)	0.81 (0.14)	Interaction	<0.0001
	Control	0.76 (0.14)	0.76 (0.11)	0.74 (0.11)	0.73 (0.09)	0.71 (13.6)	0.71 (0.13)	0.73 (0.12)		
*U*_min_, m s^−1^										
	Whole‐body heating	0.0012 (0.0004)	0.0013 (0.0003)	0.0014 (0.0006)	0.0015 (0.0007)	0.0010 (0.0016)	0.0016 (0.0008)	–	Time	0.2291
	Two‐leg heating	0.0013 (0.0002)	0.0012 (0.0004)	0.0012 (0.0005)	0.0011 (0.0003)	0.0011 (0.0003)	0.0010 (0.0004)	0.0012 (0.0004)	Condition	0.3244
	One‐leg heating	0.0012 (0.0004)	0.0013 (0.0003)	0.0013 (0.0004)	0.0012 (0.0002)	0.0011 (0.0005)	0.0012 (0.0003)	0.0014 (0.0004)	Interaction	0.9557
	Control	0.0012 (0.0004)	0.0012 (0.0004)	0.0013 (0.0003)	0.0012 (0.0003)	0.0011 (0.0003)	0.0012 (0.0002)	0.0013 (0.0002)		
*D*_max_, mm										
	Whole‐body heating	6.6 (0.7)	6.5 (0.6)	6.5 (0.6)	6.8 (0.7)	7.0 (0.7)	7.1 (0.8)	–	Time	0.0343
	Two‐leg heating	6.5 (0.5)	6.6 (0.5)	6.5 (0.5)	6.7 (0.8)	6.6 (0.6)	6.5 (0.5)	6.7 (0.5)	Condition	0.0006
	One‐leg heating	6.8 (0.7)	6.3 (0.4)	6.7 (0.8)	6.6 (0.5)	6.7 (0.7)	6.6 (0.7)	6.5 (0.7)	Interaction	0.0977
	Control	6.6 (0.6)	6.5 (0.5)	6.4 (0.3)	6.4 (0.5)	6.7 (0.4)	6.5 (0.4)	6.4 (0.5)		
*D*_min_, mm										
	Whole‐body heating	6.0 (0.7)	5.8 (0.6)	5.8 (0.6)	6.2 (0.8)	6.5 (0.8)	6.6 (0.8)	–	Time	0.0058
	Two‐leg heating	5.9 (0.5)	5.9 (0.5)	5.8 (0.5)	6.1 (0.8)	6.0 (0.6)	5.9 (0.5)	6.1 (0.5)	Condition	<0.0001
	One‐leg heating	6.0 (0.6)	5.7 (0.4)	6.0 (0.7)	5.9 (0.5)	6.0 (0.7)	6.0 (0.7)	5.9 (0.6)	Interaction	0.0694
	Control	5.9 (0.6)	5.8 (0.6)	5.7 (0.3)	5.8 (0.4)	6.0 (0.5)	5.8 (0.3)	5.7 (0.4)		

*Note*: Data are represented as the mean (SD) for eight participants. The *P*‐values were determined using two‐way ANOVA with repeated measures and the Bonferroni *post hoc* procedure. Abbreviations: *D*_max,_ maximal diameter; *D*_min_, minimal diameter; *U*_max_, maximal blood flow velocity; *U*_min_, minimal blood flow velocity.

* *P* < 0.05 vs. baseline.

^#^ *P* < 0.05 vs. control.

^†^ *P* < 0.05 vs. one‐leg heating.

^‡^ *P* < 0.05 vs. two‐leg heating.

### 3.3 Wave intensity parameters

**FIGURE 2:**
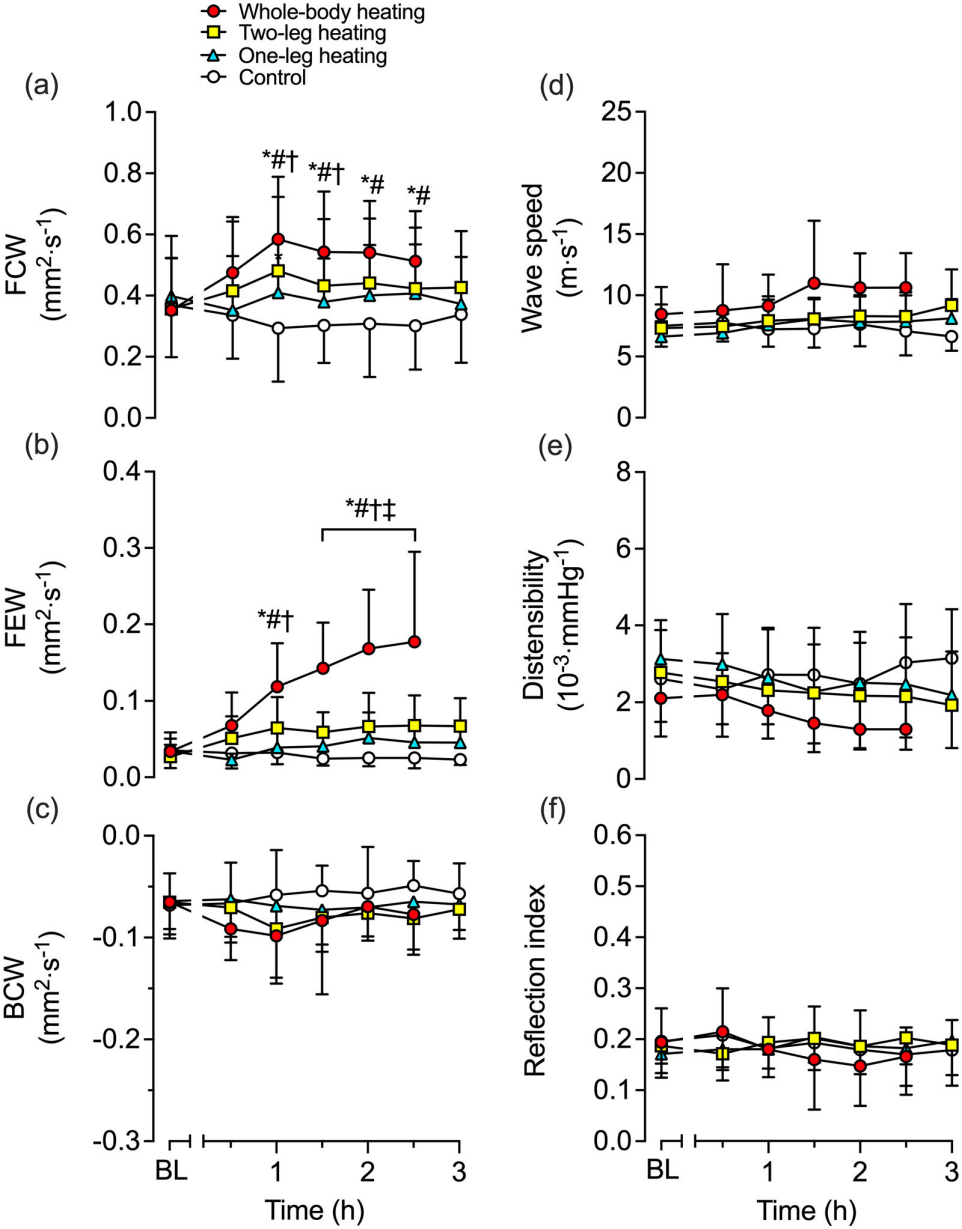
Common carotid artery (CCA) wave intensity parameters during whole‐body, two‐leg and one‐leg heating and control trials. Data are represented as the mean (SD) for eight participants. Abbreviations: BCW, backward compression wave; BL, baseline; FCW, forward compression wave; FEW, forward expansion wave. ^*^ *P* < 0.05 versus baseline; ^#^ *P* < 0.05 versus control; ^†^ *P* < 0.05 versus one‐leg heating; ^‡^ *P* < 0.05 versus two‐leg heating.

Complete profiles for WI parameters in the CCA are illustrated in Figure 2. All WIA indices remained stable over time during control, one‐leg and two‐leg heating. During whole‐body heating, FCW increased from baseline, peaking at 1 h [+0.23 (0.11) mm^2^ s^−1^; *P* < 0.0001] and remained elevated until the end of heating [+0.16 (0.17) mm^2^ s^−1^; *P* = 0.0416]. Following this increase, FCW was higher during whole‐body heating than control (time points, 1–2.5 h; all *P* < 0.0001) and one‐leg heating trials (time points, 1–1.5 h; *P *= 0.0101 and *P *= 0.0311, respectively). Despite no differences over time, FCW was different from control at 1 h during two‐leg heating (*P *= 0.0027). FEW increased progressively following 1 h of whole‐body heating until the end of heating [+0.14 (0.10) mm^2^ s^−1^; *P* < 0.0001]. FEW was higher during whole‐body heating compared with control and one‐leg heating (time points, 1–1.5 h; both *P *< 0.0001) and two‐leg heating (time points, 1.5–2.5 h; all *P* < 0.0001). No changes were observed for BCW, wave speed, compliance or reflection index during all heating protocols (*P* = 0.5422, *P* = 0.2760, *P* = 0.4891 and *P* = 0.3937, respectively).

### 3.4 Total arterial compliance and local arterial distensibility

**FIGURE 3:**
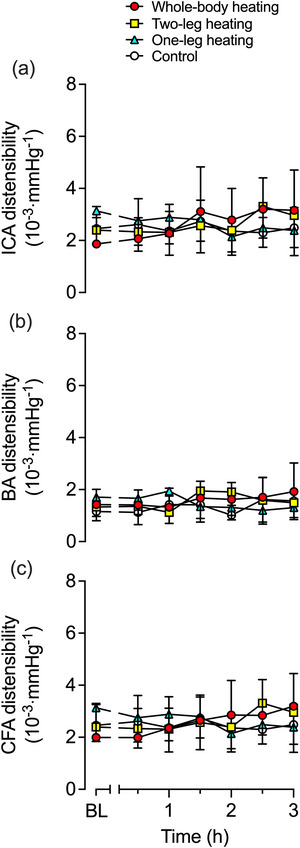
Local arterial distensibility in the major arteries perfusing the brain (ICA), forearm (BA) and leg (CFA) during whole‐body, two‐leg and one‐leg heating and control trials. Data are represented as the mean (SD) for eight participants. Abbreviations: BA, brachial artery; BL, baseline; CFA, common femoral artery; ICA, internal carotid artery.

Total arterial compliance remained unaltered over time and between protocols (*P* = 0.0866; Table 1). Likewise, at the local artery level, no significant differences were observed for CCA, ICA, BA and CFA distensibility across time × protocol (*P *= 0.1275, *P* = 0.1028, *P* = 0.1176 and *P* = 0.0601, respectively; *P*‐values for ANOVA interaction; Figure 3).

### 3.5 Associations between forward compression wave and cardiac haemodynamic parameters

**FIGURE 4:**
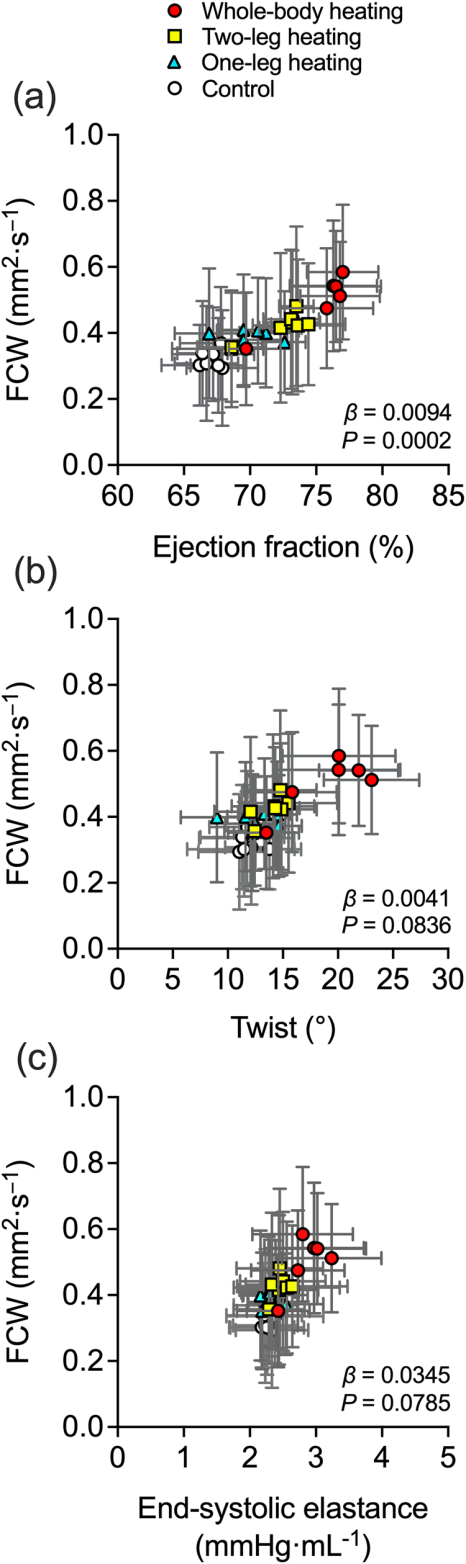
Relationships between the forward compression wave (FCW) and indexes of myocardial systolic function [left ventricular ejection fraction (a), left ventricular twist (b) and end‐systolic elastance (c)] across whole‐body, two‐leg and one‐leg heating and control trials.

Among the WI indices, only FCW demonstrated physiologically meaningful associations with the measured markers of systolic function. Specifically, the FCW (reflecting LV contractility and early systolic ejection) was significantly associated with ejection fraction [a global marker of LV systolic performance; β = 0.0094, SE = 0.0025, *t*(3.777), *P* = 0.0002; Figure 4a], but not with LV twist [a marker of systolic myocardial function; β = 0.0041, SE = 0.0023, *t*(1.741), *P* = 0.0836; Figure 4b] or end‐systolic elastance [an integrated measure of LV performance; β = 0.0345, SE = 0.0195, *t*(1.768), *P* = 0.0785; Figure 4c].

## 4 DISCUSSION

In this study, we sought to gain new insights into the haemodynamic forces underlying net increases in peripheral and systemic perfusion during lower‐limb and whole‐body hyperthermia. To accomplish this, WIA was applied in the CCA to quantify local wave speed, a marker of arterial stiffness and the inverse of distensibility, and to assess forward and backward WI parameters. In parallel, indices of local arterial distensibility and total arterial compliance, reflecting the capacity of major arteries to expand and recoil with changes in blood volume (or arterial cross‐sectional area) and pulse pressure, were measured to characterize further the mechanical contributions of the heart and downstream vasculature to changes in blood circulation. We found that FCW and FEW in the CCA were selectively augmented only during whole‐body heating, whereas the BCW, wave speed, distensibility and reflection index remained unaltered across all conditions. Likewise, distensibility of the arteries perfusing the brain (ICA), forearm (BA) and leg (CFA), in addition to total arterial compliance, were unchanged in all conditions. Collectively, these findings demonstrate that selective increases in local blood circulation during passive hyperthermia are not necessarily dependent on conduit artery mechanics or cardiac performance. Instead, they point towards haemodynamic forces generated within the peripheral microvasculature as the principal drivers of hyperthermia‐induced perfusion responses.

### 4.1 Haemodynamic forces driving blood flow during hyperthermia

The FCW and FEW in the CCA increased only during whole‐body hyperthermia, indicating enhanced LV systolic and late‐systolic dynamics. This interpretation is supported by the significant association between FCW and ejection fraction (an index of myocardial systolic function) across the four experimental trials (Figure 4a). This association was not evident when only the control or leg‐heating trials were considered. In these conditions, increases in lower‐limb and systemic blood flow and vascular conductance occurred with localized heating, without corresponding changes in CCA WI metrics or blood flow. These findings suggest that hyperaemic responses to whole‐limb or local‐limb heating are largely independent of central pressure‐driven dynamics, because they occur with preserved systolic and diastolic function and stable wave reflection (Koch Esteves et al., 2021, 2024; Watanabe et al., 2024). Our observations during lower‐limb heating are consistent with previous reports showing no differences in WIA‐derived metrics following 10 min of hot‐water immersion (Hatano et al., 2002), 30 min of upper‐ and lower‐limb heating (Athaide et al., 2023) or 3 h of one‐leg heating (Koch Esteves et al., 2021), when internal hyperthermia was mild. In contrast, the selective increases in FCW and FEW during whole‐body heating, to levels approaching heat tolerance, alongside unchanged BCW, wave speed, distensibility and reflection index, indicate enhanced central cardiac function without alterations in CCA vascular mechanics. Specifically, the rise in FCW reflects more forceful LV ejection, whereas the increase in FEW is likely to reflect augmented ventricular late‐systolic function (Feng & Khir, 2010; Hughes et al., 2013; Mynard et al., 2018; Parker & Jones, 1990). Whole‐body (but not lower‐limb) heating therefore improves systolic and diastolic cardiac function in humans (Watanabe et al., 2024). Yet, despite any augmentation of heart‐induced forward waves, regional perfusion during heat stress is not uniform: splanchnic and renal flows fall; limb, myocardial and extracranial flows rise; and cerebral perfusion is often unchanged (as in the present study) or modestly declines, depending on the arterial partial pressure of CO_2_ and heating mode (Barry et al., 2024; Chiesa et al., 2016; Crandall et al., 2008; Gibbons et al., 2020, 2021, 2020; Leaney et al., 2025; Meade et al., 2025; Minson et al., 1998; Nelson et al., 2011; Ogoh et al., 2013; Pearson et al., 2011; Rowell et al., 1969, 1970, 1969; Stöhr et al., 2011; Watanabe et al., 2024). Thus, the CCA wave intensity pattern (characterized by increased forward waves without changes in backward waves) indicates that wave reflections from the cerebral or upper‐body circulation are not enhanced. This aligns with the present observation that cerebral perfusion remains stable even as systemic flow increases by up to 6–7 L min^−1^. Such stability might reflect autoregulation mechanisms within the cerebral circulation, which helps to preserve (or at least minimize reductions in) brain blood flow in the face of widespread peripheral vasodilatation, including vasodilatation in the extracranial microvasculature. Supporting this interpretation, both forehead skin blood flow (Inoue & Shibasaki, 1996) and the diameter of skin microvessels as small as ∼30 µm (Costa et al., 2025) increase substantially in response to passive and local heating, respectively. This suggests that the extracranial microvasculature undergoes marked vasodilatation even when cerebral circulation is maintained or slightly reduced.

### 4.2 Circulatory control during hyperthermia

Passive properties of conduit arteries, such as arterial distensibility, together with active modulation by vascular smooth muscle tone, determine arterial diameter and thereby contribute to peripheral blood flow control (Green et al., 2017; Laughlin et al., 2012; Nichols et al., 2022). In anaesthetized mice, heating has recently been shown to alter vascular tone in skeletal muscle, gut, brain and skin arteries, while, paradoxically, increasing arterial and venous blood flow in the radial muscle branch artery and an adjacent forelimb vein despite a reduction in vessel diameter (Phan et al., 2025). These findings suggest that changes in vascular tone and arterial diameter might be accompanied by alterations in arterial distensibility, which could affect tissue perfusion and venous return during passive heating. Contrary to this notion, no statistically significant changes were detected in diameter (∆*D*), pulse pressure or calculated distensibility of the CCA, ICA, BA and CFA during lower‐limb and whole‐body heating. This absence of change contrasts with the arterial responses we previously reported during graded exercise (incremental semi‐recumbent cycling to 70% peak power output), where CCA distensibility decreases by 79% from resting baseline while the peak and area of the FCW and FEW progressively increase (up to +452% and 315%; +718% and 900%, respectively), together with a gradual decline in the peak and area of the BCW (down to −700% and −390%). These changes were associated with significant elevations in ∆*D* (+58%), ∆*U* (+93%), pulse pressure (+78%), wave speed (+121%) and CCA blood flow (+32%), all of which returned to baseline during recovery (Pomella et al., 2018). Although direct WIA comparisons between exercise and passive hyperthermia are lacking, a plausible explanation for these divergent responses is that whole‐body exercise elicits substantially greater sympathoadrenal activation and elevations in circulating vasoconstrictors, such as noradrenaline (20–50 vs. 2–4 nmol L^−1^, respectively), angiotensin II, vasopressin and endothelin (Gagnon et al., 2015; González‐Alonso & Calbet, 2003; Ichinose et al., 2008; Low et al., 2011; McMorris et al., 2006; Niimi et al., 1997; Saito et al., 1993; Trangmar et al., 2014, 2017, 2014), which could increase arterial stiffness by augmenting vasoconstrictor activity.

A principal aim of the present study was to determine whether heat‐mediated elevations in kinetic energy of blood returning to the heart during limb and whole‐body hyperthermia are attributable to region‐specific alterations in arterial properties. The absence of changes in arterial distensibility (CCA, ICA, BA and CFA) and total arterial compliance observed in the present study suggests that the mechanical properties of the arterial tree remain stable during thermal stress to levels approaching maximal heat tolerance. This stability further indicates that thermal hyperaemia arises predominantly within the downstream microcirculation rather than in the conduit arteries. Multiple lines of evidence support the notion that local mechanisms play a central role in controlling blood flow within specific vascular territories. In mice, myogenic tone in peripheral arteries is modulated by temperature via the thermosensitive channels TRPV1 and TRPM4, thereby influencing regional tissue perfusion (Phan et al., 2025). In humans, regional leg heating selectively increases blood velocity, flow and shear rate within heated tissues, without altering perfusion pressure (Koch Esteves et al., 2021, 2024). Likewise, whole‐body heating augments femoral arterial and venous flow in humans (Chiesa et al., 2016), and local heating stimuli can increase blood velocity and flow even in the absence of central cardiac activity (Li & Pollack, 2023; Manteuffel‐Szoege, 1960, 1969). Importantly, local heat, opposing electrical and ionic changes, and concentration gradients along the capillary lumen have all been shown to contribute to self‐driven flow (Li & Pollack, 2020). This might represent a pathway through which heat is converted into kinetic energy, thereby increasing blood velocity and flow independently of the haemodynamic forces generated by the pressure gradient maintained by the pumping function of the heart. The present findings are consistent with prior reports demonstrating that central and peripheral arterial stiffness and distensibility remain largely unaltered during acute whole‐body (Ganio et al., 2011; Moyen et al., 2016; Schlader et al., 2019) and localized limb hyperthermia (Athaide et al., 2023; Hatano et al., 2002; Koch Esteves et al., 2021). Significantly, the present study extends those observations by demonstrating that local distensibility and total arterial compliance (hence the mechanical properties of the major conduit arteries supplying the head, brain, forearm and leg) remain unchanged despite substantial heat‐induced increases in blood flow, particularly in the CFA.

The present graded experimental design (control, one‐leg, two‐leg and whole‐body heating) reveals that only systemic thermal stress elicits measurable haemodynamic alterations in wave patterns. The absence of changes in cardiac waves during localized lower‐limb heating suggests that selective increases in blood flow are not attributable solely to augmented cardiac force transmission. Elevated tissue temperature induces rheological and biochemical adjustments that reduce blood viscosity and promotes vasodilatation in the networks of the small arteries and arterioles within specific regions, as discussed above. These include: increased red cell deformability and axial dispersion, which enhance capillary perfusion (Çinar et al., 2001; Manteuffel‐Szoege, 1960, 1969; Pinho et al., 2016); reductions in blood viscosity and flow resistance, which lower vascular impedance (Çinar et al., 2001; Lim et al., 2010; Shin et al., 2004; Snyder, 1971); and enhanced release of vasodilatory mediators, such as erythrocyte‐derived ATP and endothelial nitric oxide (Etulain et al., 2011; Kalsi & González‐Alonso, 2012; Kalsi et al., 2017; Kellogg et al., 1999; Minson et al., 2001). These local vasodilatory mechanisms counteract sympathetically and humorally mediated vasoconstriction, which is typically heightened during systemic heat stress but attenuated during isolated leg heating (Cui et al., 2004; Gagnon et al., 2015; Keller et al., 2006; Low et al., 2011; Niimi et al., 1997; Takahashi et al., 2011). Hyperthermia thus modulates vascular tone differentially across vascular beds (Phan et al., 2025) and heating modalities, as thermoregulatory and rheological mechanisms influence adrenergic and humoral restraint (Rowell, 1990; Trangmar & González‐Alonso, 2025). This integrative balance supports perfusion in heat‐dissipating regions (extracranial, legs, arms and torso; Costa et al., 2025; Inoue & Shibasaki, 1996; Koch Esteves et al., 2021, 2024) while modulating venous return, probably explaining the selective increases in blood flow occurring without changes in cardiac wave intensity patterns during passive lower‐limb hyperthermia. Collectively, these observations support the concept that passive hyperthermia enhances blood circulation in specific regions predominantly through peripheral and microvascular mechanisms while preserving the elastic and wave transmission properties of the central arterial network.

### 4.3 Methodological considerations and limitations

This study assessed WI metrics in the CCA, providing important insight into vascular dynamics and central cardiac function. However, WIA in the CCA does not fully capture haemodynamic interactions and arterial wall properties across the systemic circulation. Large arteries differ in structure, function and responses to stressors, such as passive hyperthermia (Larson et al., 2021; Nichols et al., 2022), hence the extrapolation of CCA findings to other regions must be done with caution. A practical limitation was the relatively low spatial resolution of ultrasound images in the ICA, BA and CFA, which prevented reliable WIA in these arteries. As a result, we were unable to compare regional differences in wave dynamics and arterial wall mechanics directly. To address this limitation, in part, we applied complementary approaches: (1) total arterial compliance as an integrated measure of systemic behaviour; and (2) and regional estimates of arterial distensibility derived from pressure and vessel diameter. It should be noted that this latter approach also has its own limitations, because upper‐arm blood pressure was used to calculate pulse pressure rather than directly measured local pulse pressure at each arterial site. Nonetheless, both approaches demonstrated unchanged CCA distensibility across time and protocols, despite yielding slightly different absolute values, reflecting the nature of the parameters measured (instantaneous, forward‐wave stiffness vs. cardiac cycle‐average, systemic estimate). Together, these findings provide useful surrogate insights into vascular responses when WIA was not feasible. Looking forwards, ultra‐high‐frequency ultrasound offers a promising way to overcome current technical limitations by enabling more reliable WIA in smaller and deeper arteries and in veins (Izzetti et al., 2021). This advance would facilitate comprehensive mapping of regional arterial and venous responses to hyperthermia, supporting further development of current computetional models (Zhang et al., 2026). Additionally, future work should recruit more heterogeneous populations, spanning both sexes, a wider age range and clinical groups with altered vascular function (e.g., hypertension, arterial stiffness and venous insufficiency). Such diversity would improve generalizability and help to characterize how hyperthermia influences large vessel haemodynamics across the population.

A potential limitation of the present study is that no a priori sample size calculation was performed for the WI‐derived outcomes, because these analyses were conducted as part of a secondary analysis within a broader investigation of human circulatory control during passive hyperthermia (Watanabe et al., 2024). Consequently, the sample size (*n* = 8) was determined by the primary physiological outcomes of the parent study, for which power calculations based on blood flow responses indicated sufficient sensitivity but were not tailored to WIA metrics. To address this limitation, effect magnitudes and prospective sample‐size estimates were evaluated. FCW responses to one‐ and two‐leg heating were small in comparison to control, with projected sample sizes suggesting limited physiological relevance (Athaide et al., 2023; Koch Esteves et al., 2021). In contrast, FEW exhibited large, consistent effects compared with the control and one‐ and two‐leg heating conditions, with estimates indicating adequate power in the present cohort. Alongside the absence of changes in related vascular variables (e.g., CCA diameter, mean diameter, wave speed, distensibility, blood flow and vascular conductance) during lower‐limb(s) heating and the significant FCW and FEW responses observed during whole‐body heating, these findings suggest that the experimental design was capable of detecting physiologically meaningful effects in forward‐travelling waves when present.

## 5 CONCLUSION

This investigation demonstrated that, in the CCA, the WI parameters FCW and FEW increased only during whole‐body heating that approached maximal heat tolerance, whereas BCW, wave speed, distensibility and reflection index remained unchanged across all conditions. Correspondingly, no notable changes in local ICA, BA and CFA distensibility or total arterial compliance were observed, despite significant increases in leg and systemic blood flow across all hyperthermic protocols. Collectively, these findings indicate that selective increases in blood flow during passive hyperthermia are largely independent of conduit artery mechanics and cardiac performance and are instead likely to be driven by haemodynamic forces within the peripheral microvasculature. External heating is an effective and practical intervention for enhancing or activating these physiological mechanisms.

## AUTHOR CONTRIBUTIONS

This study is part of a comprehensive investigation focusing on the control of blood circulation during hyperthermia (Watanabe et al., 2024). José González‐Alonso and Nuno Koch Esteves conceived and designed the study with respect to WIA and the total arterial compliance responses to leg(s) and whole‐body hyperthermia. Kazuhito Watanabe, José González‐Alonso and Nuno Koch Esteves were involved in the data collection. Nuno Koch Esteves analysed the regional blood flow data and, together with Francesca R. Cavallo, analysed the WIA, guided by feedback from Ashraf W. Khir. José González‐Alonso and Kazuhito Watanabe calculated the total arterial compliance data and prepared the figures and tables. Nuno Koch Esteves and José González‐Alonso prepared the manuscript, which was later revised with input from all the authors. All authors revised the manuscript for important intellectual content. All authors approved the final version of the manuscript and agree to be accountable for all aspects of the work in ensuring that questions related to the accuracy or integrity of any part of the work are appropriately investigated and resolved. All persons designated as authors qualify for authorship, and all those who qualify for authorship are listed.

## CONFLICT OF INTEREST

None declared.

## Data Availability

The data that support the findings of this study are available from the first and last authors upon reasonable request.
